# A new technique to study nutrient flow in host-parasite systems by carbon stable isotope analysis of amino acids and glucose

**DOI:** 10.1038/s41598-022-24933-9

**Published:** 2023-01-19

**Authors:** Tobias Hesse, Milen Nachev, Shaista Khaliq, Maik A. Jochmann, Frederik Franke, Jörn P. Scharsack, Joachim Kurtz, Bernd Sures, Torsten C. Schmidt

**Affiliations:** 1 Instrumental Analytical Chemistry, University of Duisburg-Essen, Universitätsstr. 5, 45141 Essen, Germany; 2 Aquatic Ecology, University of Duisburg-Essen, Universitätsstr. 5, 45141 Essen, Germany; 3 Centre for Water and Environmental Research, University of Duisburg-Essen, Universitätsstr. 5, 45141 Essen, Germany; 4 Institute for Evolution & Biodiversity, University of Münster, Hüfferstr. 1, 48149 Münster, Germany; 5 Present Address: Thünen Institute of Fisheries Ecology, Herwigstr. 31, 27572 Bremerhaven, Germany; 6 Present Address: Bavarian State Institute of Forestry, Hans-Carl-Von-Carlowitz-Platz 1, 85354 Freising, Germany

**Keywords:** Freshwater ecology, Stable isotope analysis

## Abstract

Stable isotope analysis of individual compounds is emerging as a powerful tool to study nutrient origin and conversion in host-parasite systems. We measured the carbon isotope composition of amino acids and glucose in the cestode *Schistocephalus solidus* and in liver and muscle tissues of its second intermediate host, the three-spined stickleback (*Gasterosteus aculeatus*), over the course of 90 days in a controlled infection experiment. Similar linear regressions of δ^13^C values over time and low trophic fractionation of essential amino acids indicate that the parasite assimilates nutrients from sources closely connected to the liver metabolism of its host. Biosynthesis of glucose in the parasite might occur from the glucogenic precursors alanine, asparagine and glutamine and with an isotope fractionation of − 2 to – 3 ‰ from enzymatic reactions, while trophic fractionation of glycine, serine and threonine could be interpreted as extensive nutrient conversion to fuel parasitic growth through one-carbon metabolism. Trophic fractionation of amino acids between sticklebacks and their diets was slightly increased in infected compared to uninfected individuals, which could be caused by increased (immune-) metabolic activities due to parasitic infection. Our results show that compound-specific stable isotope analysis has unique opportunities to study host and parasite physiology.

## Introduction

Parasites are ubiquitously present in our environment and contribute high amounts of biomass to ecosystems, rivaling that of top predators^1,2^. They are major parts of ecological food webs^3–5^ and can account for substantial energy and carbon transfer^6,7^. A key difference between parasites and predators is that parasites interact with less victims (host) during a single life stage, and this interaction does not necessarily result in the death of its host^8^. The nutrients that parasites acquire are therefore derived from fewer sources, and since they can occupy specific tissues or organs, this limitation can be extended in many cases to, e.g., only host muscle or blood as dietary sources. Some parasites inhabiting the hosts gut can also feed on undigested gut content and their nutrients are not even strictly host derived^9^. Because of these different interactions and the fact that parasites are difficult to observe and sample in nature, ecologists struggle to incorporate them in their studies, leading to false assumptions in terms of structure, density, resistance to stressors and energy flow of environments^10^.

One well-studied host-parasite model system is the cestode *Schistocephalus solidus* and its specific intermediate host, the three-spined stickleback (*Gasterosteus aculeatus). S. solidus* is an endoparasite with a complex life cycle, in which the stickleback represents the second intermediate host harboring the plerocercoid stage inside of its body cavity. The plerocercoid avoids the host’s immune system and grows substantially in size^11,12^. During this time, huge amounts of glucose are built and stored as glycogen inside the parasite, which are used later for maturation and reproduction^13,14^ and the cestode manipulates the stickleback’s behavior^15,16^ to increase transmission to the final host, usually a fish-eating bird. Based on techniques for culturing the parasite in vitro and infecting sticklebacks experimentally, *S. solidus* has been extensively used to investigate host parasite-interactions such as immune response, reproductive development, effects on host behavior and movement abilities^11,16–21^.

A recent review highlights the importance and usefulness of stable isotope analysis (SIA), which has been recognized as a powerful tool over the last decade to gain insights into the energy and nutrient exchange between parasite and host^22^. In SIA, isotope signatures of an element are measured as the ratio of heavy to light isotopes and referenced to an international standard of known isotopic composition, leading to the widely used δ-value, expressed in per mil (‰). Biochemical reactions tend to discriminate against the heavier isotope, resulting in isotope fractionations where, in comparison to the external supply, the instantaneously built product is isotopically depleted (the ratio of heavy to light isotope is lower) and the remaining substrate gets isotopically enriched (the ratio of heavy to light isotope is higher)^23^. The initial carbon isotope signatures in environments are fixed by primary producers, mainly plants and microorganisms, but also vary according to the exact mechanisms employed by these organisms. In water bodies, e.g., δ^13^C values can differentiate between pelagic and littoral sources because baseline values of littoral food webs are isotopically ^13^C-enriched compared to pelagic food webs^24^. When nutrients then traverse through a food chain, their overall isotope signatures tend to increase per level of trophic transmission, which is most apparent for nitrogen with an average increase in δ^15^N by 3–4 ‰ of consumer tissue compared to its diet, but also to a smaller extent for carbon with an average increase of < 1 ‰^25^. The exact metabolic reason for trophic fractionation is still not fully understood, but the most likely explanation is that conversion and catabolism of nutrients for energy production leads to mineralization and excretion of isotopically depleted end-products (e.g., CO_2_ or urea), whereas the remaining isotopically enriched substrates are then measured in tissue samples. It is worth noting, however, that variations in trophic fractionation can occur between species and a universal trophic fractionation factor is most likely not adequate^26^. Furthermore, *de-novo* synthesis of compounds like non-essential amino acids (NEAA) from other cell compartments can significantly contribute to their carbon isotope signature and lead to different patterns of trophic fractionation depending on dietary composition^27–31^.

Measurements can be either done from whole sample materials in bulk stable isotope analysis (BSIA) or, due to advances in recent years in the coupling of isotope ratio mass spectrometers (IRMS) to chromatographic separation techniques, from individual compounds in compound specific isotope analysis (CSIA). There are major advantages of using isotope ratios of single constituents compared to bulk tissue. One important shortcoming of BSIA is that the isotope signature of bulk tissue can be confounded by variations in baseline values across space and time by primary producers, which complicates the interpretation of isotope values on higher trophic levels. CSIA can overcome this shortcoming by measuring isotope signatures of different compound classes. The isotope signature of, e.g., essential amino acids (EAA), due to their nature to traverse food chains isotopically mostly unchanged, then represents baseline values of primary producers and dietary sources in a higher trophic organism and can be directly compared to the isotope signature of NEAA to estimate origin of resources and nutrient utilization on different diets in a variety of research fields^27,32–41^. This might be especially useful for the study of host-parasite interactions. A comparative literature-based analysis of 101 host-parasite pairs revealed a large range of trophic discrimination factors for both nitrogen (− 6.7 to + 9.0 ‰) and carbon (− 8.2 to 6.5 ‰) using BSIA^42^, showing unusual fractionation patterns with both enriched and depleted isotope signatures of parasites compared to host organisms.

While the stable isotope signatures of nitrogen usually show significant stepwise enrichment per trophic transfer and are therefore used to calculate trophic positions, the carbon isotope signature changes little per trophic transfer and is used to determine original sources of dietary carbon^43^, although catabolism and metabolic reactions can still induce significant isotope fractionation especially for individual compounds like amino acids^27,29,37,44^. The transfer and conversion of nutrients within host-parasite systems is not fully known and CSIA can give valuable insights and elucidate information hidden to regular BSIA. The aim of this study was to (1) determine the nutrient source of the parasite within the host organism, (2) investigate the origin of glucose storages for maturation and (3) compare trophic fractionation between infected and uninfected control sticklebacks from an earlier study^45^. We therefore measured the carbon stable isotope signature of thirteen individual AAs and glucose of the cestode *S. solidus* in addition to muscle and liver tissue of its second intermediate host, the three-spined stickleback, in a controlled infection experiment over the course of 90 days post infection (dpi). CSIA of nitrogen has already been used to determine trophic positions of parasites more accurately^46^, and we are the first to apply CSIA of carbon to examine nutrient flow and conversion in host-parasite relationships. Our overall goal is to outline possible metabolic pathways, which can be studied using CSIA, and stimulate future research.

## Results

### Changes in AA δ^13^C values over 90 days after infection

δ^13^C values of AAs overall ranged between − 25 and 0 ‰ on the VPDB scale, with patterns of lowest δ^13^C values between − 25 and – 20 ‰ for Phe, Tyr and Val and highest δ^13^C between − 10 and 0 ‰ for Ser and Gly (Table S1).

Adjusted coefficients of determinations (Adj. R^2^) of linear regressions from dietary δ^13^C values between 30 and 90 dpi were higher than 0.73 (DF = 7) for any individual AA. Slopes of regression curves were between − 0.069 and − 0.028 ‰/dpi with significant differences from zero (one-way ANOVA, DF = 1, 7, p ≤ 0.002), showing negative linear relationships between δ^13^C values of AAs and dpi (Table S2) in the used food source.

In parasite and liver tissues, analysis of linear regression showed that δ^13^C values of the NEAAs Ala, Asx, Glx, Tyr and the EAAs Arg and Lys decreased over time (one-way ANOVA of regression slopes against zero, DF = 1, 13, p < 0.01), with significant negative slopes between − 0.02 and − 0.06 ‰/dpi (DF = 13, Table S2). Comparing the regression slopes of individual AAs between sample tissues showed that Ala, Asx, Glx, Tyr, Arg and Lys were significantly different between tissues (*F* test, DF = 2, 39, p < 0.01, Table S3). For Ala, Arg and Lys, those differences were observed between host liver and muscle tissue. Differences in linear regressions between host muscle and parasite tissue were seen for Asx, Glx and Tyr (pairwise *F* test, DF = 1, 26, p ≤ 0.001, Table S3) and for Ser between parasite and liver tissue (*F* test, DF = 1, 26, p = 0.009, Table S3).

### δ^13^C values of Glucose

The supply of glycogen that *S. solidus* acquired had a carbon isotope signature between − 15 and − 18 ‰ (Table S1), with a linear decrease of − 0.060 ± 0.007 ‰/dpi (Adj. R^2^ = 0.84, Table S2) in the regression slope between 30 and 90 dpi, which was significantly different from zero (one-way ANOVA, DF = 1, 13, p ≤ 0.001, Table S3). AAs with a comparable linear decrease in their δ^13^C values are Ala and Glx in host liver (− 0.054 ± 0.006 and − 0.044 ± 0.011 ‰/dpi) and Asx, Glx and Tyr in parasite tissue (− 0.048 ± 0.00, − 0.050 ± 0.008 and − 0.045 ± 0.007 ‰/dpi). Ala, Asx and Glx additionally had similar carbon isotope signatures between − 10 and – 15 ‰, with glucose in parasite tissue being isotopically ^13^C-depleted by − 2 to – 3 ‰ in comparison.

### Trophic fractionation between parasite and host tissues

**Figure 1:**
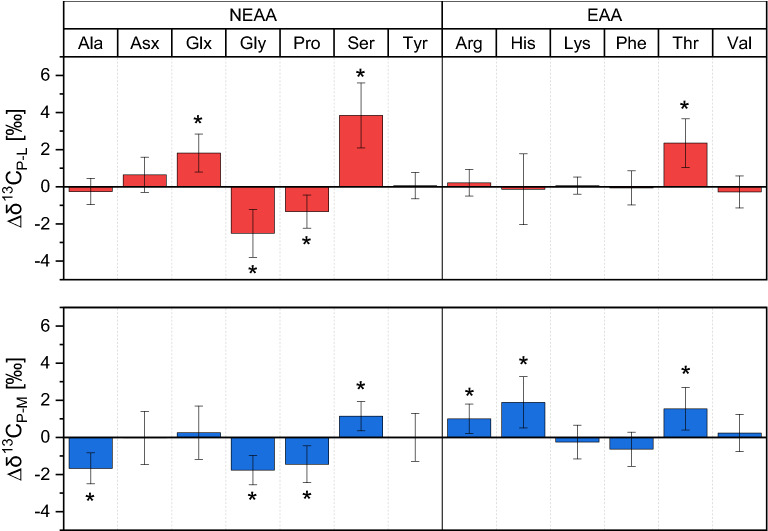
Average trophic fractionation (Δδ^13^C ± SD in ‰, n = 15) of individual AAs between parasite (P), host liver (L) and muscle (M) tissue over 90 dpi. Asterisks (*) indicate significant differences of average Δδ^13^C values from zero (two-sided *t* tests, DF = 14, p < 0.01). Trophic fractionation of EAAs between parasite and host liver was very low except for Thr, whereas significant Δδ^13^C_P-M_ values between the parasite and muscle tissue of the EAAs Arg, His and Thr were observed. Δδ^13^C_P-L_ values were negative for the NEAAs Gly and Pro and positive for Ser and Glx. Note that trophic fractionation of Asx and Glx between the parasite and muscle tissue seemed low and not significant, but regression slopes of δ^13^C values from Asx and Glx over time were very different between the two tissues (compare Table S3) and are therefore not directly comparable.

We calculated average trophic fractionation (Δδ^13^C_P-M/L_ ± SD) between parasite and host muscle/liver tissue over 90 dpi (n = 15, Fig. 1). Δδ^13^C_P-M/L_ values were tested with two-sided* t* tests against zero and significant differences are marked with an asterisk (DF = 14, p < 0.01). Average values and statistical data are reported in Table S4. Trophic fractionation was not measurable for almost all EAAs except Thr between parasite and liver tissue, whereas Δδ^13^C_P-M_ values of Arg and His were additionally significant between parasite and muscle. NEAAs showed more often fractionation between parasite and liver tissue, with Glx and Ser being isotopically ^13^C-enriched, whereas Gly and Pro were ^13^C-depleted in the parasite compared to host liver. Note that although Δδ^13^C_P-M_ values of Asx and Glx between parasite and muscle tissue were close to zero, both NEAAs showed very different changes of δ^13^C values over time points and are therefore not directly comparable. There was no universal trend of either positive or negative trophic fractionation between parasite and host liver, with Asx, Ser and Thr having positive Δδ^13^C_P-L_ values, whereas Gly and Pro showed the opposite trend of negative trophic fractionation.

### Trophic fractionation patterns between infected and uninfected sticklebacks

**Figure 2:**
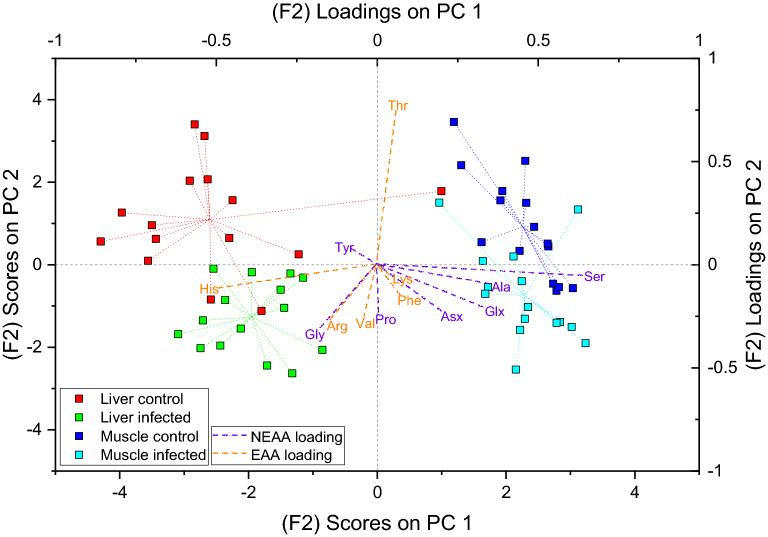
Multivariate analysis of Δδ^13^C_Fish-Diet_ values shows higher trophic fractionation of most individual AAs in liver and muscle tissue of infected sticklebacks compared to uninfected control individuals on the same diet. PC1 of the first factor (tissue) from ASCA analysis separates muscle and liver tissue of the infected and control individuals, whereas PC2 shows separate clusters of infected and control liver and muscle tissues.

Δδ^13^C_Fish-Diet_ values of individual AAs between infected and uninfected control sticklebacks did not show any differences between liver or muscle tissues in univariate analysis except for Thr (Table S5), which was significantly lower in the liver of infected fish (− 1.0 ± 1.8 ‰) compared to uninfected liver samples (0.8 ± 1.0 ‰). We therefore performed ASCA on Δδ^13^C_Fish-Diet_ values of AAs as a more powerful multivariate approach. Time points had the highest effect (30.78%) on system variance, while tissue type accounted for 26.78% (Table S6). To investigate fractionation patterns between infected and uninfected individuals, we will focus on tissue type (factor 2). A biplot with sample scores and AA loadings on PC1 and PC2 (Fig. 2) shows separate clusters of liver and muscle samples, separated by differences in their Δδ^13^C_Fish-Diet_ values by mainly His and Ser with loadings of − 0.5 and 0.64, respectively. Liver scores on PC1 are negative and center around mean values of − 2.6 ± 1.3 for uninfected and − 2.0 ± 0.7 for infected samples, whereas muscle scores are positive with mean values of 2.3 ± 0.6 for both uninfected and infected individuals. PC2, however, shows differences in sample scores between infected and uninfected samples for both liver and muscle tissue. Uninfected samples on PC2 have positive scores for liver (1.1 ± 1.3) and muscle (0.9 ± 1.3) tissue, whereas infected samples have negative scores for liver (− 1.3 ± 0.9) and muscle (− 0.7 ± 1.1) tissue. The separation is not as clear as between liver and muscle tissues and there are some overlapping samples. Eleven out of the thirteen measured AAs have negative loadings on PC2, with only Thr and Tyr having positive loadings. The influence of His, Ser, Tyr and Lys might be miniscule due to their loadings being close to zero on PC2, but the results show a universal trend of tissues from infected sticklebacks having higher trophic fractionation values of carbon for most AAs except Thr, which shows the opposite trend of lower Δδ^13^C values in both muscle and liver tissue compared to uninfected individuals on the same diet.

## Discussion

We measured the carbon isotope signature of 13 individual AAs of the cestode *S. solidus* and liver and muscle tissue of its second intermediate host, the three-spined stickleback. Although we sampled over the course of 120 days, we only considered samples up to 90 dpi to have a linear shift of dietary δ^13^C values, which showed a sudden increase after 120 days in comparison to the linear decrease over the first 90 days. The origin of the dietary isotope shift in the range of ~ 3–4 ‰ over time remains unclear, as described in our previous work^45^, but could possibly be attributed to different batches of mosquito larvae used throughout the feeding experiment. Host liver and parasite tissue showed similar linear decreases over 90 dpi for six of the thirteen measured AAs in contrast to more constant isotope signatures of muscle tissue over time.

The sticklebacks used in this infection experiment were reared in parallel to the individuals described in our previous studies^45^. The dietary isotope signature of AAs changed over the course of the experiment and caused a significant shift of δ^13^C-values in the liver tissue of uninfected control sticklebacks, whereas δ^13^C-values in muscle tissue remained unaffected. Analogous to uninfected sticklebacks and to earlier bulk isotope studies investigating carbon isotope turnover in different tissue types^47–49^, the liver of infected fish also adapted quickly to the dietary isotope shift. The liver is an essential organ for nutrient metabolism and serves as a modifying and communicating link between the digestive tract of an organism and its peripheral tissues^50^. The parasite, on the other hand, absorbs nutrients from the surrounding body cavity of its host through the tegument^51^. It has been shown in studies using BSIA of carbon and nitrogen that passively feeding parasites do not follow typical trophic fractionation patterns, which makes a direct comparison of bulk isotope signatures between parasite and fish tissues difficult for source allocation. Instead of isotope enrichment, as it is expected in a predator–prey relationship, some gutless helminths such as cestodes and acanthocephalans exhibit lower δ-values for carbon and nitrogen compared to their hosts^52–55^. It is speculated that these parasites feed on reprocessed metabolites, which are lighter in their isotope signature and might originate from the host’s liver and gut metabolism (Nachev et al.^52^, Gilbert et al.^55^). Recent research on the tapeworm *Schyzocotyle acheilognathi*, which is an adsorptive feeder inside of fish intestines, also points towards nutrients being derived from liver metabolites^54^. Although *S. solidus* does not live inside the gut of its host and has no direct access to liver metabolites, its presence inside the body cavity between liver and gut could enable the parasite to adsorb nutrients from liver excretes. The observed dietary isotope shifts in fish diet and associated change of carbon isotope signatures in stickleback liver and parasite tissue over time support this idea. Comparing the regression slopes showed that especially the isotope signatures of Asx and Glx over time in the parasite do not match the general trend of constant isotope signatures in muscle tissue, and the change over time of parasitic Asx and Glx instead is identical to changes observed in liver tissue. This could be interpreted as a close connection between the nutrients that *S. solidus* assimilates and the hosts liver metabolism. Interestingly, some AAs in parasite and host tissues did not show an overall negative shift over time in their isotope signatures, which was the case for Gly, Pro, Ser, His, Phe and Thr. Slopes of linear regression were either close to zero or showed large variation, such that statistical tests would not indicate significant differences. For example, average regression slopes of Gly in liver and parasite tissue over 90 dpi were − 0.021 ‰/dpi for both tissues, but liver tissues had higher uncertainties resulting in no statistically significant difference from zero in contrast to parasite samples. It appears that these AAs do not follow dietary trends on shorter time scales, which in case of NEAAs could indicate *de-novo* synthesis from other compounds like lipids, carbohydrates and other AAs, but could also stem from catabolism and nutrient conversion associated with isotope fractionation.

The ability of CSIA to identify nutrient sources hinges on differences in isotope signatures between those sources, which are subsequently transferred to higher trophic organisms and visible in compounds which show no or only minor trophic fractionation. Comparing trophic fractionation of EAAs between parasite and host tissue is consequently only useful for resource allocation when there are significant differences in the isotope signatures of EAAs between liver and muscle tissue. Many EAAs did not show such differences between muscle and liver tissue except for His, which had higher δ^13^C-values in the liver (Table S1). Unusual fractionation patterns of His between diets and uninfected sticklebacks were already mentioned and discussed in our earlier study^45^, where His not only showed high trophic fractionation between uninfected sticklebacks and diets but also higher trophic fractionation in liver compared to muscle tissue. The same trends were also seen in this study for infected sticklebacks. Average Δδ^13^C values of His and other EAAs except Thr between parasite and host liver are close to zero, whereas Δδ^13^C values between parasite and muscle tissue show significant differences for His and additionally Arg. Overall, the similar δ^13^C shifts of AAs over time and lower trophic fractionation of EAAs between parasite and liver tissue suggests that nutrient absorption and assimilation in *S. solidus* takes place through liver metabolites and not through sources derived from muscle tissue of its host. We will therefore subsequently focus on isotopic fractionation between parasite and liver tissue to investigate metabolic pathways for nutrient conversion, parasitic growth and the buildup of glycogen storages for growth and reproduction.

Glycogen is the major macronutrient for cestodes and the glycogen content in *S. solidus* plerocercoids can reach up to 50% of dry weight^13^. It might be unlikely that the parasite is able to acquire all of its glycogen directly from the host and biosynthesis of glucose from other nutrients may be necessary. Interestingly, half of the proteins in the secretome of the parasite show enriched functions for glycolytic processes and gluconeogenesis in addition to cell division^16^, which suggests that biosynthesis of glucose might not only happen inside the parasite from assimilated sources, but also outside in the secretome directly from host nutrients. The changes in δ^13^C values of glucose in the parasite over time were similar to those of the NEAAs Ala, Asx and Glx, with a constant offset between -2 to -3 ‰. This would fit the idea of enzymatic reactions discriminating against the heavier isotope and inducing isotopic fractionation to lower δ^13^C values in the built product. Furthermore, the carbon isotope signature of Ala in liver and parasite tissue showed some striking similarities. Ala itself is an important precursor for gluconeogenesis and can be directly converted to pyruvate via deamination, which is mainly used by organisms to cycle AAs and carbohydrates between tissues^56^. This would indicate that the parasite has access to the alanine-glucose cycle of its host, which results in identical isotope signatures of alanine in parasite and host liver because extensive amounts of nutrients are transferred this way without noticeable fractionation.

The AAs Gly, Pro, Ser and Thr were less affected by the isotope shift over time resulting in no significant slopes in linear regression over 90 dpi. Thr is an essential AA and must be taken up through diets by higher organisms. It has been shown that animals can convert significant amounts of Thr to Gly^57^, which is itself convertible to Ser and vice versa^58^. Thr can also be catabolized extensively to yield Gly and acetate during the growth of single-cell parasites^59^. The enriched carbon isotope signatures of Thr in the parasite could therefore indicate extensive conversion of Thr to produce Gly, which would again match the idea of enzymatic reactions discriminating against the heavier isotope^23^. This could further explain the lower carbon isotope signatures of Gly in parasite tissue compared to both fish muscle and liver, as metabolic processes of Thr catabolism would yield a product which is isotopically depleted compared to the substrate, in addition to the already lower carbon isotope signature of Thr itself. Unusual stable isotope fractionation of Thr has been reported on several occasions^46,60–63^, where Thr was isotopically ^15^N depleted in consumers compared to diets. This goes against the established idea of increasing δ^15^N values per trophic level and a possible explanation of the widely observed Thr ^15^N-depletion includes an inverse isotope effect of the enzyme threonine deaminase preferably removing ^15^N-amino groups, possibly because ^15^N forms stronger bonds in the formation and interchange of intermediate Schiff bases^63^. However, the conversion of Thr to Gly goes by a different mechanism and is catalyzed by the enzyme threonine dehydrogenase, which oxidizes Thr to 2-amino-3-ketobutyrate in a first step^64^. Although carbon and nitrogen show different tendencies of isotope fractionation for Thr, both phenomena are not contradicting each other and could be a result of different enzymatic reactions. It is interesting to see different fractionation patterns of Thr in relation to metabolic activities, especially since a study on ^15^N fractionation of amino acids in a host-parasite system, including negative trophic fractionation of Thr, has been recently published^46^.

Gly can be used to produce Ser by reversing the Gly biosynthesis pathway using serine hydroxymethyl transferase. However, carbon isotope signatures of Ser in the parasite are enriched in ^13^C compared to muscle and liver throughout the experiment, which does not indicate extensive conversion of Gly to Ser. A more dominant pathway for Gly catabolism in animals and plants is the Gly cleavage system to produce 5, 10-methylene-H4 folate, which is an important C1 donor in biosynthesis processes^65^. The enriched carbon isotope signature of Ser in the parasite can therefore be interpreted in a similar way to Thr, where Ser is utilized leading to enriched isotope signatures compared to diets. It is worth mentioning that Ser was subject to coelution with the much more abundant Glx during chromatography and the isotope signatures of Ser therefore might be compromised to some degree (see “Materials and Methods”). However, coelution of Ser and Glx were most apparent in muscle samples and less in liver and parasite (Fig. S5B–D), so the effect of Ser coeluting with Glx might be miniscule to the discussion of Δδ^13^C values between parasite and liver.

The important connection of Ser and Gly to one-carbon metabolism for cell growth and proliferation has been described in literature^66–68^ and would be necessary for the parasite to grow substantially in size inside of the stickleback. Biosynthesis of Ser from other compounds might be unlikely to result in higher δ^13^C, as Ser typically has the highest carbon isotope signature of all AAs^27,29,33,34,44^, possibly due to its high conversion rates in organisms and its metabolic importance for cell proliferation^67^. As seen in our results, the carbon isotope signature of carbohydrates such as glucose in parasite tissue is also depleted compared to Ser, which makes conversion of glucose into Ser unlikely to result in enriched Ser isotope signatures and lipids are typically even more ^13^C-depleted^69^.

Comparing Δδ^13^C values of AAs between infected and uninfected sham-exposed sticklebacks on the same diet in a multivariate analysis showed a general pattern of higher Δδ^13^C values in muscle and liver tissue of infected individuals for almost all AAs except Thr and Tyr. Both classes of NEAAs and EAAs contributed to the separation of infected and uninfected tissue scores on PC2 from ASCA, indicating that differences are caused by a general shift in AA metabolism, independent of their class. Parasites are bound to have severe impacts on host metabolism. It was shown that infected sticklebacks have higher respiration rates by oxygen consumption than uninfected individuals and that the additional oxygen consumption of the parasite does not cover these discrepancies^70^. The nutrients that the parasite accumulates are on account of host organisms, which have to fuel parasitic growth in addition to its own survival and energy demands. An increase in trophic fractionation of NEAAs could be explained by increased biosynthesis to maintain cell homeostasis, as recent research showed that biosynthesis of AAs from precursor molecules like pyruvic acids or α-ketoglutaric acid can also lead to ^13^C-enriched NEAAs like Gly, Ala and Glx^71^. However, this is not the case for EAAs which cannot be synthesized. Increased catabolism of EAAs, like earlier mentioned for Thr in parasite tissue, could be another reason, but there is to date no scientific study that investigated small trophic fractionation values of EAAs due to metabolic reactions. It is certainly true that these compounds remain mostly conservative to trophic fractionation, but there are still metabolic pathways they are involved in that should induce fractionation, even if not to the same extent as for NEAAs.

Furthermore, parasitic infection causes a response of the host’s immune system to fight and remove the infection, which is an ongoing burden on the host’s energy metabolism. T-Cell activation, e.g., is a metabolically demanding process and requires nutrients such as Ala, Glx, Ser and Arg^72^. Host-parasite systems such as *G. aculeatus* and *S. solidus* showed upregulation of host immune response during later infection stages, when the parasite was already established^12^ and would explain their increased fractionation patterns in infected sticklebacks to fuel an ongoing immune reaction. Furthermore, the highest negative loading on PC2 for differentiation of Δδ^13^C values between infected and uninfected sticklebacks were observed for Gly, which is an important neurotransmitter, anti-inflammatory and immunomodulatory agent^73^ preventing apoptosis, sepsis and endotoxic shock. Immunometabolism is a recently emerging field in medical research to understand auto-immune disorders^74^. Helminth parasites can modulate the immunometabolism of their hosts and are recognized as models to study immunometabolic principles^18,75,76^. It is interesting to see that not only nutrient metabolism, but possibly also immunometabolism might be a subject of investigation for CSIA, giving new prospects for future research.

## Conclusion

We could provide first insights into the carbon isotope signatures and fractionation of individual AAs and glucose between the cestode *S. solidus* and its second intermediate host, the three-spined stickleback. The parasite likely gets most of its nutrients from sources closely related to the liver metabolism of its host, which should be considered as comparing whole body or muscle tissue of host and parasite might give misleading information. The carbon isotope signatures of glucose could be directly measured after hydrolysis due to the high amounts present in parasite tissue, which makes it an ideal candidate along with amino acids for future studies preferably using artificially enriched materials to quantify energy and nutrient flow. We highlighted two promising areas in amino acid and glucose metabolism in that regard, namely biosynthesis of glucose from glucogenic AA precursors, especially Ala, Asx and Glx, and conversion of Thr, Ser and Gly for one carbon metabolism. Higher trophic fractionation of AAs in infected sticklebacks compared to uninfected sham-exposed individuals on the same diet indicate increased catabolism of AAs to either fight off the infection through an ongoing immune response and/or to sustain parasitic growth and cell homeostasis. Our results show that CSIA of carbon bears additional prospects in the study of host-parasite physiology and immunometabolism.

## Materials and methods

### Infection experiment

Three-spines sticklebacks and parasites were maintained and treated in accordance with the local animal welfare authorities and the EU Directive 2010/63/EU for animal experiments and were approved by the ‘State Agency for Nature, Environment and Consumer Protection’ (LANUV) of North Rhine Westphalia. The infection and breeding experiments were carried out in accordance with the local veterinary and animal welfare authorities under the project number 87.51.04.2010.A297. All methods were further carried out in compliance with the ARRIVE guidelines (https://arriveguidelines.org/).

Three-spined sticklebacks were collected from a brook (Ibbenbürener Aa, 52°17′33.11"N, 7°36′46.48"E, North-West Germany) and held in 14 L tanks (VewaTech, Germany) at 18 °C with recirculating water. Light/dark cycles were set to 15/9 h and sticklebacks were fed daily with red mosquito larvae (Chironomidae).

Larval parasites were produced by in vitro breeding of adults according to established methods^77,78^, replacing the bird host by in vitro culture allowing for reproduction of the adult cestodes. The released eggs were washed, stored in tap water at 4 °C and incubated for three weeks at 20 °C in the dark. The hatching of free living coracidia was initiated by illumination for 3 h, followed by 8 h in the dark and again illumination for 2 h. Single coracidia were transferred to wells containing one single copepod (*Macrocyclops albidus*) in 2 mL tap water. Copepods were checked after two weeks for the presence of *S. solidus* procercoids.

Sticklebacks for the infection experiments were eight months old and tagged individually with visible implant elastomer tags (Northwest Marine Technologies, USA) three weeks before parasite exposure. 144 sticklebacks in total were divided into twelve 14 L tanks, each containing a group of 12 individuals, and fasted 72 h prior to exposure before they were transferred to glass jars filled with 300 mL tank water. On the next day, 3 sticklebacks per group were exposed to an uninfected copepod (sham-exposed) and 9 sticklebacks were exposed to an infected copepod, containing a three-week-old procercoid. The water was sieved after 24 h to confirm ingestion of the copepod and sticklebacks were then returned to the water tanks.

36 Sticklebacks were sampled 30, 60, 90 and 120 dpi each and fasted for 72 h prior to sampling. Ingestion of an infected copepod does not always lead to parasitic infection (exposed but not infected), and out of the 108 sticklebacks exposed to an infected copepod, 74 individuals were itself infected with the parasite after ingestion. We analyzed five infected and five uninfected (sham-exposed) individuals in this study on each time point. Although sampling was done over a period of 120 days, we only considered samples up to 90 dpi after the start of the experiment. An explanation why the last sampling date was removed is given in the data analysis section. Anesthetization was done with MS 222 (Sigma Aldrich, USA) and sticklebacks were subsequently killed by decapitation. The body cavity was opened and *S. solidus* plerocercoids were removed and washed with MilliQ water. Liver and muscle samples of sticklebacks were collected and all samples were stored at − 20 °C. Five infected sticklebacks (liver and muscle tissue) and their respective parasites were randomly chosen per time point for CSIA, which is an identical number of individuals as in our previous study on sham-exposed sticklebacks.

### Sample preparation and CSIA via LC-IRMS

Hydrolysis and sample analysis of individual AAs were carried out analogously to our previous study^45^. In brief, approximately 5 mg sample material were hydrolyzed with 2.5 mL 6 M hydrochloric acid (> 99%, Alfa Aesar, Kandel, Germany) at 110 °C in 5 mL PTFE vials (CEM GmbH, Kamp-Lintfort, Germany) for 24 h. The hydrolysate was filtered (0.2 µm PTFE filter), evaporated to dryness and reconstituted in 1 mL distilled water. CSIA was carried out on a Dionex Ultimate 3000 HPLC Pump (Thermo Fisher Scientific, Bremen, Germany) coupled to an Isolink Interface and Delta V Advantage mass spectrometer (Thermo Fisher Scientific, Bremen, Germany) with a mixed mode cation exchange column (Primesep A, 2.1 mm ID, 250 mm L, 5 µm particle size) from SIELC. Chromatographic separation of AA in-house standards and sample materials is shown in the supporting information (Fig. S5A–D). The method can separate 14 individual standard AAs (Fig. S5A), although measurable quantities of Met in sample materials were not observed. Separation and measurement of sample AAs can occasionally be compromised by partial coelution of matrix interferences and other AAs, which is a common problem in CSIA of AAs via LC-IRMS and caused by large peak widths due to the design of the interface and difficult separation conditions. Although partly coeluting peaks can still be measured accurately with careful peak integration and background detection^79^, results of Asx and Ser might be influenced to some degree in our case. Asx coeluted with high amounts of matrix components eluting early from the column especially in liver and parasite samples (Fig. S5C,D), and Ser partly coeluted with the much higher Glx peak especially in muscle samples (Fig. S5B).

Using liquid chromatography allows for separation and measurement of underivatized AAs in contrast to gas chromatography, where derivatization is required and complicates isotope analysis^80^. This allowed for recent advances in the field of CSIA by enabling position-specific stable isotope analysis of AAs, multi-dimensional HPLC and the coupling of IRMS and high-resolution mass spectrometry (HRMS)^81–83^. Referencing and normalization of isotope data is also simplified by avoiding kinetic isotope effects during derivatization and the introduction of additional carbon to target analytes, which overall increases systematic errors and measurement uncertainty in GC-IRMS. We used sixteen in-house amino acid standards with a purity of > 98% (Alfa Aesar, Kandel, Germany) and seven certified international reference materials (L-Alanine, L-Glutamic acid, USGS 64, USGS 66, L-Phenylalanine, L-Proline and L-Valine, ordered from Arndt Schimmelmann, Department of Earth and Atmospheric Sciences at Indiana University, Bloomington, IN, USA) for normalization of our data. The sixteen in-house standards were normalized individually on a vario Pyro cube Elemental Analyzer coupled to an Isoprime100 Mass Spectrometer (Elementar Analysensysteme GmbH, Langenselbold, Germany) with the seven certified reference materials in a multi-point calibration to acquire the true isotope signature of in-house standards. A mix of the sixteen in-house standards was then regularly injected throughout measurement periods (Fig. S5A) and we occasionally injected a mix of the seven certified reference materials to assure long-term stability of our system. Note that we only used thirteen of the in-house AA standards for our analysis, since Iso and Leu completely coeluted and Met was not measurable in any sample material due to low abundance. Measured isotope signatures of in-house standards were pooled, e.g., before and after the filament of the ion source had to be changed, and individually used to calculate the true isotope signature of sample AAs by using the difference between true and measured δ^13^C value of each in-house standard (compound-specific correction) with a gaussian error propagation. This way, we were able to follow the identical treatment procedure^84^ of sample and reference material to avoid the problem of differing oxidation efficiencies, which can occur between substances during wet-chemical oxidation^85^.

Sample preparation for glucose analysis was carried out by acid hydrolysis of 5–10 mg sample material in 5 mL of 1.1 M sulfuric acid at 120 °C for 1 h^86^. The hydrolysate was filtered (0.2 µm PTFE filter, WICOM, Germany) and neutralized to pH 6–7 by adding Ca(OH)_2_ and CaSO_4_. The samples were stored overnight at − 4 °C for precipitation and filtered again. Chromatographic separation of glucose was achieved with an ion-exchange column (Rezex™ RCM-Monosaccharide, 7.8 × 300 mm, 8 µm, Phenomenex, Germany) and pure Milli-Q water as eluent. Flow rate was set to 400 µLmin^−1^ and the column was heated to 80 °C. While direct stable isotope measurements of glucose from muscle or liver tissue were not feasible due to low concentrations of glucose in combination with little amounts of sample materials, the abundance of glucose in the parasite was sufficient for direct injections of 10 µL sample volume and δ^13^C measurements via LC-IRMS. If this method can be used for stable isotope measurements of glucose from higher amounts of muscle and liver materials could be a subject for future studies, possibly in combination with appropriate pre-concentration techniques to overcome the problem of limited instrument sensitivity and to remove matrix interferences. This was outside the scope of this study but would further improve the application of CSIA to study host-parasite interactions.

### Data analysis

Isotope data was analyzed using Excel from Microsoft Office 365 ProPlus (Microsoft, Redmond, Washington, USA), Origin 2019 version 9.60 (OriginLab, Northampton, Massachusetts, USA) and Matlab R2021a (MathWorks Inc., Natick, Massachusetts, USA) with the PLS_Toolbox suite (Eigenvector Research Inc., Manson, WA). Isotope data are reported as mean δ^13^C values for each AA and glucose on individual time points on the VPDB scale in per mill (‰) with its corresponding standard deviation (SD) in the supplementary material (Table S1, Fig. S1). Data was tested for normality with Kolmogorov–Smirnov-Tests and by Brown-Forsythe tests (α = 0.05) to check for equality of group variances.

We used linear fits and *F* tests between slopes of the regression line to investigate changes in δ^13^C values of individual AAs and tissues on a time series. *F* tests were conducted between all sample tissues and additionally pairwise to investigate differences between individual sample tissues (liver vs. muscle, muscle vs. parasite, etc.). We measured a noticeable shift in dietary δ^13^C values of individual AAs over time in the same experimental design for uninfected fish samples^45^, but the shift was not linear over the complete period (Fig. S2). δ^13^C values of dietary samples decreased by approximately − 2‰ between 30 and 60 and 30–90 dpi, but the last shift between 90 and 120 dpi was positive and δ^13^C values increased on average by + 2‰. To approximate data by a linear fit for fish and parasite tissue, we removed the last time point from the analysis and only analyzed changes in δ^13^C values between the first 90 dpi.

We calculated average trophic fractionation between parasite and host liver/muscle tissue over 90 dpi (n = 15) and tested the significance with two-sided* t* tests against zero (DF = 14, α = 0.01). We chose to also exclude the last sampling date at 120 dpi so we can consider the slopes of linear regression during the discussion, as trophic fractionation of AAs with differences in regression slopes between tissues are not directly comparable.

We further compared trophic fractionation of liver and muscle tissue from infected to uninfected control sticklebacks in a multivariate approach using ANOVA simultaneous component analysis (ASCA). Data of uninfected sticklebacks and dietary samples were taken from our earlier study using the same experimental design^45^. Tissues and time points were set as independent variables (factors) and AA Δδ^13^C_Fish-Diet_ values of infected and uninfected sticklebacks as variables. Multivariate data analysis was used because the differences in trophic fractionation were small but consistent for many AAs, which makes a multivariate approach more powerful compared to a univariate analysis by, e.g., regular ANOVA. The analysis was performed without data pre-processing and with 1000 permutations.

## Supplementary Information

Supplementary Information.

## Data Availability

The datasets generated during and/or analysed during the current study are available in the Figshare repository: https://doi.org/10.6084/m9.figshare.21070909.v1.
