# Supplementary material for: A new technique to study nutrient flow in host-parasite systems by carbon stable isotope analysis of amino acids and glucose
Source: Sci Rep. 2023 Jan 19;13:1054. doi: 10.1038/s41598-022-24933-9 (PMC9852285; doi:10.1038/s41598-022-24933-9)
Supplement: Supplementary file 1 — Supplementary Information. [file 41598_2022_24933_MOESM1_ESM.pdf]

# A new technique to study nutrient flow in host-parasite systems by carbon stable isotope analysis of amino acids and glucose

Authors:

Tobias Hesse[1], Milen Nachev[2,3], Shaista Khaliq[1], Maik A. Jochmann*[,1,3], Frederik Franke[4,6], Jörn P. Scharsack[4,5], Joachim Kurtz[4], Bernd Sures[2,3], Torsten C. Schmidt[1,3]

Affiliations:

1. Instrumental Analytical Chemistry, University of Duisburg-Essen, Universitätsstr. 5, 45141 Essen, Germany
2. Aquatic Ecology, University of Duisburg-Essen, Universitätsstr. 5, 45141 Essen, Germany
3. Centre for Water and Environmental Research, University of Duisburg-Essen, Universitätsstr. 5, 45141 Essen, Germany
4. Institute for Evolution & Biodiversity, University of Münster, Hüfferstr. 1, 48149 Münster, Germany
5. Present address: Thünen Institute of Fisheries Ecology, Herwigstr. 31, 27572 Bremerhaven, Germany
6. Present address: Bavarian State Institute of Forestry, Hans-Carl-von-Carlowitz-Platz 1, 85354 Freising, Germany

# Supporting Information

**Table S1:** Average carbon isotope signatures and SD (n = 5) of individual AAs for liver, muscle and parasite tissue after 30, 60, 90 and 120 DPI in ‰ on the VPDB scale.

| Tissue | DPI | Ala | | Asp | | Glu | | Gly | | Pro | | Ser | | Glucose | |
|---|---|---|---|---|---|---|---|---|---|---|---|---|---|---|---|
| | | Avg | SD | Avg | SD | Avg | SD | Avg | SD | Avg | SD | Avg | SD | Avg | SD |
| Liver | 30 | -12.3 | 0.6 | -11.5 | 0.8 | -11.1 | 1.1 | -6.4 | 0.8 | -13.6 | 0.9 | -3.6 | 1.0 | - | - |
| | 60 | -14.6 | 0.4 | -12.1 | 1.1 | -13.4 | 0.5 | -6.3 | 1.4 | -13.8 | 0.9 | -4.5 | 1.4 | - | - |
| | 90 | -15.5 | 0.5 | -13.6 | 0.7 | -14.3 | 1.1 | -7.3 | 1.1 | -14.6 | 1.1 | -5.1 | 1.0 | - | - |
| | 120 | -15.4 | 0.5 | -14.4 | 1.4 | -13.4 | 0.8 | -7.7 | 1.0 | -13.3 | 1.5 | -5.4 | 1.1 | - | - |
| Muscle | 30 | -12.3 | 0.5 | -11.8 | 0.7 | -11.2 | 0.5 | -7.1 | 0.7 | -14.3 | 0.8 | -1.5 | 0.2 | - | - |
| | 60 | -12.8 | 0.7 | -11.6 | 0.7 | -11.3 | 0.9 | -7.7 | 0.6 | -14.1 | 1.2 | -2.0 | 0.7 | - | - |
| | 90 | -13.0 | 0.4 | -11.9 | 0.7 | -11.6 | 0.6 | -7.4 | 0.8 | -13.3 | 1.0 | -1.5 | 0.8 | - | - |
| | 120 | -13.6 | 0.6 | -12.3 | 0.5 | -11.9 | 0.7 | -8.7 | 0.7 | -13.6 | 0.4 | -3.3 | 0.6 | - | - |
| Parasite | 30 | -13.1 | 0.2 | -10.1 | 0.6 | -9.2 | 0.7 | -9.1 | 1.1 | -15.2 | 1.3 | -0.8 | 0.7 | -14.6 | 0.9 |
| | 60 | -15.0 | 0.5 | -12.2 | 0.3 | -11.7 | 0.2 | -8.9 | 0.4 | -15.4 | 0.6 | -0.8 | 0.6 | -16.5 | 0.3 |
| | 90 | -15.1 | 0.1 | -13.1 | 0.4 | -12.5 | 0.8 | -9.6 | 0.9 | -15.4 | 1.1 | 0.0 | 0.9 | -18.1 | 0.6 |
| | 120 | -14.8 | 0.3 | -13.5 | 0.5 | -13.0 | 0.9 | -10.0 | 0.7 | -15.5 | 0.9 | -0.2 | 0.4 | -17.5 | 0.3 |

| Tissue | DPI | Arg | | His | | Lys | | Phe | | Thr | | Tyr | | Val | |
|---|---|---|---|---|---|---|---|---|---|---|---|---|---|---|---|
| | | Avg | SD | Avg | SD | Avg | SD | Avg | SD | Avg | SD | Avg | SD | Avg | SD |
| Liver | 30 | -16.8 | 0.4 | -7.9 | 0.7 | -14.8 | 0.3 | -23.9 | 0.6 | -14.0 | 0.7 | -21.2 | 0.4 | -19.7 | 0.5 |
| | 60 | -16.8 | 0.3 | -9.8 | 1.1 | -15.7 | 0.4 | -24.0 | 0.8 | -12.0 | 1.1 | -21.5 | 0.6 | -20.4 | 1.0 |
| | 90 | -18.6 | 0.6 | -9.4 | 0.6 | -16.8 | 0.7 | -25.1 | 0.7 | -14.2 | 0.6 | -23.0 | 0.5 | -21.6 | 1.0 |
| | 120 | -19.5 | 0.7 | -9.5 | 0.9 | -17.8 | 0.6 | -25.5 | 0.8 | -12.4 | 1.0 | -23.3 | 0.9 | -22.3 | 0.8 |
| Muscle | 30 | -18.0 | 0.8 | -11.2 | 1.0 | -15.3 | 0.6 | -23.6 | 0.7 | -11.6 | 1.1 | -22.0 | 0.8 | -20.9 | 0.8 |
| | 60 | -18.4 | 0.6 | -10.6 | 0.4 | -15.5 | 0.5 | -23.8 | 0.6 | -13.3 | 0.4 | -21.7 | 0.9 | -21.4 | 1.3 |
| | 90 | -18.1 | 0.4 | -11.4 | 0.6 | -15.5 | 0.5 | -23.9 | 0.3 | -12.9 | 0.9 | -21.9 | 0.5 | -20.9 | 1.1 |
| | 120 | -18.5 | 0.7 | -12.2 | 0.5 | -16.1 | 0.4 | -24.7 | 0.5 | -12.6 | 0.9 | -22.1 | 0.4 | -21.4 | 0.3 |
| Parasite | 30 | -16.6 | 0.4 | -9.6 | 1.1 | -14.8 | 0.4 | -24.4 | 0.7 | -11.2 | 1.1 | -20.7 | 0.6 | -20.4 | 1.0 |
| | 60 | -17.2 | 0.3 | -9.0 | 1.2 | -15.6 | 0.3 | -24.0 | 0.8 | -10.9 | 0.8 | -21.9 | 0.6 | -20.9 | 0.7 |
| | 90 | -17.8 | 0.6 | -8.9 | 0.9 | -16.7 | 0.7 | -24.8 | 0.5 | -11.2 | 0.9 | -22.9 | 0.8 | -21.2 | 0.5 |
| | 120 | -18.1 | 0.5 | -8.9 | 1.0 | -16.7 | 0.5 | -24.4 | 0.9 | -10.4 | 0.7 | -22.9 | 0.5 | -21.6 | 0.9 |

**Table S2:** Parameters (y-intercept, slope and adjusted coefficient of determination) of linear regression for dietary, liver, muscle and parasite tissue. Standard deviations are given in brackets and p-values are drawn from one-way ANOVA of regression slopes, indicating significant differences from zero. Significance levels for one-way ANOVA and F-tests was set to 0.05 and significant differences are marked bold.

| AA | Tissue | Y-Intercept [‰] | | Slope [‰/dpi] | | p-value (slope = 0) | Adj. $R^2$ |
|---|---|---|---|---|---|---|---|
| Ala | Diet | -9.5 | (0.4) | -0.069 | (0.006) | **0.000** | 0.947 |
| | Liver | -10.9 | (0.4) | -0.054 | (0.006) | **0.000** | 0.835 |
| | Muscle | -11.9 | (0.4) | -0.013 | (0.006) | 0.035 | 0.244 |
| | Parasite | -12.5 | (0.4) | -0.032 | (0.006) | **0.000** | 0.673 |
| Asx | Diet | -9.3 | (0.2) | -0.052 | (0.004) | **0.000** | 0.950 |
| | Liver | -10.9 | (0.6) | -0.029 | (0.009) | **0.007** | 0.402 |
| | Muscle | -12.1 | (0.4) | 0.004 | (0.006) | 0.572 | -0.050 |
| | Parasite | -8.9 | (0.4) | -0.048 | (0.007) | **0.000** | 0.786 |
| Glx | Diet | -9.8 | (0.4) | -0.047 | (0.006) | **0.000** | 0.877 |
| | Liver | -10.4 | (0.8) | -0.044 | (0.011) | **0.001** | 0.523 |
| | Muscle | -11.1 | (0.5) | -0.005 | (0.007) | 0.518 | -0.042 |
| | Parasite | -8.2 | (0.5) | -0.050 | (0.008) | **0.000** | 0.753 |
| Gly | Diet | -4.6 | (0.2) | -0.046 | (0.004) | **0.000** | 0.946 |
| | Liver | -5.3 | (1.0) | -0.021 | (0.015) | 0.171 | 0.073 |
| | Muscle | -6.7 | (0.4) | -0.009 | (0.007) | 0.250 | 0.031 |
| | Parasite | -7.8 | (0.4) | -0.021 | (0.006) | **0.006** | 0.408 |
| Pro | Diet | -11.5 | (0.5) | -0.036 | (0.007) | **0.002** | 0.743 |
| | Liver | -12.8 | (0.5) | -0.018 | (0.007) | 0.029 | 0.265 |
| | Muscle | -14.7 | (0.7) | 0.016 | (0.011) | 0.182 | 0.066 |
| | Parasite | -15.4 | (0.6) | 0.000 | (0.009) | 0.966 | -0.077 |
| Ser | Diet | 1.0 | (0.3) | -0.060 | (0.005) | **0.000** | 0.945 |
| | Liver | -2.9 | (1.1) | -0.025 | (0.017) | 0.171 | 0.073 |
| | Muscle | -1.8 | (0.5) | -0.001 | (0.008) | 0.894 | -0.075 |
| | Parasite | -1.3 | (0.6) | 0.009 | (0.009) | 0.321 | 0.005 |
| Tyr | Diet | -18.4 | (0.4) | -0.036 | (0.007) | **0.002** | 0.748 |
| | Liver | -20.4 | (0.3) | -0.026 | (0.005) | **0.000** | 0.681 |
| | Muscle | -22.2 | (0.5) | 0.004 | (0.009) | 0.691 | -0.063 |
| | Parasite | -19.1 | (0.5) | -0.045 | (0.007) | **0.000** | 0.731 |

| | | | | | | | |
|---|---|---|---|---|---|---|---|
| Arg | Diet | -16.1 | (0.3) | -0.037 | (0.005) | **0.000** | 0.888 |
| | Liver | -15.8 | (0.5) | -0.024 | (0.007) | **0.005** | 0.430 |
| | Muscle | -17.8 | (0.5) | -0.007 | (0.008) | 0.397 | -0.017 |
| | Parasite | -16.0 | (0.3) | -0.019 | (0.005) | **0.002** | 0.505 |
| His | Diet | -14.3 | (0.5) | -0.037 | (0.008) | **0.002** | 0.734 |
| | Liver | -7.6 | (0.6) | -0.026 | (0.010) | 0.023 | 0.288 |
| | Muscle | -10.8 | (0.5) | -0.006 | (0.007) | 0.455 | -0.030 |
| | Parasite | -9.8 | (0.6) | 0.008 | (0.011) | 0.459 | -0.031 |
| Lys | Diet | -12.8 | (0.3) | -0.050 | (0.005) | **0.000** | 0.926 |
| | Liver | -14.1 | (0.3) | -0.029 | (0.005) | **0.000** | 0.744 |
| | Muscle | -15.5 | (0.3) | 0.002 | (0.004) | 0.729 | -0.067 |
| | Parasite | -13.9 | (0.3) | -0.029 | (0.004) | **0.000** | 0.750 |
| Phe | Diet | -20.6 | (0.2) | -0.049 | (0.004) | **0.000** | 0.955 |
| | Liver | -23.6 | (0.5) | -0.012 | (0.008) | 0.127 | 0.106 |
| | Muscle | -23.4 | (0.4) | -0.006 | (0.006) | 0.387 | -0.014 |
| | Parasite | -24.0 | (0.6) | -0.004 | (0.009) | 0.629 | -0.057 |
| Thr | Diet | -9.2 | (0.2) | -0.053 | (0.004) | **0.000** | 0.962 |
| | Liver | -12.5 | (0.7) | -0.012 | (0.010) | 0.266 | 0.024 |
| | Muscle | -11.6 | (0.6) | -0.027 | (0.009) | 0.014 | 0.336 |
| | Parasite | -11.3 | (0.7) | -0.002 | (0.010) | 0.837 | -0.073 |
| Val | Diet | -17.6 | (0.4) | -0.028 | (0.005) | **0.001** | 0.770 |
| | Liver | -18.8 | (0.5) | -0.027 | (0.008) | **0.005** | 0.431 |
| | Muscle | -20.8 | (0.6) | -0.003 | (0.009) | 0.754 | -0.068 |
| | Parasite | -20.4 | (0.5) | -0.009 | (0.006) | 0.161 | 0.080 |
| Glucose | Diet | - | | - | | - | - |
| | Liver | - | | - | | **-** | - |
| | Muscle | - | | - | | **-** | - |
| | Parasite | -12.8 | (0.5) | -0.060 | (0.007) | **0.000** | 0.840 |

**Table S3:** P-values from F-tests of regression slopes over 90 days after infection between parasite and host tissue. Tests were conducted between all tissues (DF = 2, 39) and pairwise (L vs M, L vs P, M vs P, DF = 1, 26) and significant differences are marked bold ($p < 0.01$). Differences were found for Ala, Asx, Glx, Tyr, Arg and Lys, although slopes for Ala, Arg and Lys only differed between liver and muscle tissue. Asx, Glx and Tyr, in addition, also showed significant differences in regression slopes between muscle and parasite tissue, but not between parasite and liver. The only difference between parasite and liver were observed for Ser, but those were close to the significance level of 0.01 and the overall F-test was above this limit with a p-value of 0.013.

| | AA | F-test | L vs M | L vs P | M vs P |
|---|---|---|---|---|---|
| **NEAA** | Ala | **0.000** | **0.000** | 0.030 | 0.016 |
| | Asx | **0.000** | **0.006** | 0.198 | **0.000** |
| | Glx | **0.000** | **0.001** | 0.795 | **0.000** |
| | Gly | 0.813 | 0.554 | 0.680 | 0.840 |
| | Pro | 0.111 | 0.042 | 0.406 | 0.211 |
| | Ser | 0.013 | 0.059 | **0.009** | 0.245 |
| | Tyr | **0.001** | **0.004** | 0.390 | **0.001** |
| **EAA** | Arg | **0.005** | **0.004** | 0.198 | 0.028 |
| | His | 0.031 | 0.109 | 0.017 | 0.239 |
| | Lys | **0.004** | **0.004** | 0.350 | 0.017 |
| | Phe | 0.262 | 0.128 | 0.219 | 0.872 |
| | Thr | 0.395 | 0.323 | 0.832 | 0.158 |
| | Val | 0.046 | 0.024 | 0.119 | 0.277 |

**Table S4:** Average trophic fractionation ($\Delta\delta^{13}C$) of AAs between parasite and liver/muscle tissue of host in ‰ over 90 days after infection. Standard deviations are given in brackets (n = 15). Significant differences of $\Delta\delta^{13}C$ values from zero were tested individually with two-sided t-tests (DF = 14, α = 0.01) and are marked bold.

| | AA | Parasite-Liver | | | | Parasite-Muscle | | | |
|---|---|---|---|---|---|---|---|---|---|
| | | $\Delta\delta^{13}C$ (‰) | | t-test | P-Value | $\Delta\delta^{13}C$ (‰) | | t-test | P-Value |
| **NEAA** | **Ala** | -0.3 | (0.7) | -1.41 | 0.180 | -1.7 | (0.8) | -7.73 | **0.000** |
| | **Asx** | 0.6 | (0.9) | 2.62 | 0.020 | 0.0 | (1.4) | -0.10 | 0.923 |
| | **Glx** | 1.8 | (1.0) | 6.87 | **0.000** | 0.3 | (1.4) | 0.69 | 0.500 |
| | **Gly** | -2.5 | (1.3) | -7.55 | **0.000** | -1.8 | (0.8) | -8.69 | **0.000** |
| | **Pro** | -1.3 | (0.9) | -5.81 | **0.000** | -1.4 | (1.0) | -5.65 | **0.000** |
| | **Ser** | 3.8 | (1.7) | 8.50 | **0.000** | 1.1 | (0.8) | 5.63 | **0.000** |
| | **Tyr** | 0.1 | (0.7) | 0.32 | 0.751 | 0.0 | (1.3) | 0.00 | 0.997 |
| **EAA** | **Arg** | 0.2 | (0.7) | 1.16 | 0.266 | 1.0 | (0.8) | 4.89 | **0.000** |
| | **His** | -0.1 | (1.9) | -0.28 | 0.787 | 1.9 | (1.4) | 5.28 | **0.000** |
| | **Lys** | 0.1 | (0.5) | 0.53 | 0.608 | -0.3 | (0.9) | -1.08 | 0.299 |
| | **Phe** | -0.1 | (0.9) | -0.27 | 0.794 | -0.6 | (0.9) | -2.69 | 0.017 |
| | **Thr** | 2.3 | (1.3) | 6.94 | **0.000** | 1.5 | (1.1) | 5.19 | **0.000** |
| | **Val** | -0.3 | (0.9) | -1.25 | 0.233 | 0.2 | (1.0) | 0.92 | 0.372 |

**Table S5:** Comparison of trophic fractionation between infected sticklebacks and sham exposed control sticklebacks from our previous study. P-values indicate significant differences between either liver and muscle tissue of infected and uninfected tissues on a significance level of 0.01. Differences were not observed for almost all AAs except Thr between liver tissues, which was significantly depleted in host liver tissue compared to control liver samples.

| | $\Delta\delta^{13}C_{Lc-D}$ | | $\Delta\delta^{13}C_{Lh-D}$ | | p-value | $\Delta\delta^{13}C_{Mc-D}$ | | $\Delta\delta^{13}C_{Mh-D}$ | | p-value |
|---|---|---|---|---|---|---|---|---|---|---|
| **Ala** | -0.9 | (1.4) | -0.4 | (0.9) | 0.258 | 0.8 | (1.5) | 1.0 | (1.6) | 0.821 |
| **Asx** | -0.7 | (1.0) | 0.1 | (1.0) | 0.054 | 0.4 | (1.2) | 0.7 | (1.6) | 0.538 |
| **Glx** | -0.8 | (1.2) | -0.3 | (1.0) | 0.054 | 0.6 | (1.1) | 1.3 | (1.2) | 0.132 |
| **Gly** | 0.2 | (1.5) | 0.7 | (1.4) | 0.356 | -0.7 | (1.1) | -0.1 | (1.3) | 0.129 |
| **Pro** | -0.6 | (1.5) | -0.2 | (1.1) | 0.410 | -0.8 | (0.9) | -0.1 | (1.7) | 0.220 |
| **Ser** | -2.1 | (1.2) | -1.6 | (1.3) | 0.348 | 1.0 | (1.6) | 1.1 | (1.6) | 0.840 |
| **Tyr** | -1.0 | (0.8) | -1.5 | (0.6) | 0.063 | -1.7 | (1.0) | -1.5 | (1.2) | 0.513 |
| **Arg** | 0.3 | (0.6) | 0.9 | (0.6) | 0.019 | -0.3 | (0.9) | 0.1 | (1.1) | 0.272 |
| **His** | 8.0 | (1.2) | 7.5 | (1.1) | 0.245 | 5.1 | (1.5) | 5.4 | (1.1) | 0.496 |
| **Lys** | 0.1 | (0.8) | 0.1 | (0.6) | 0.955 | 0.1 | (1.0) | 0.4 | (1.3) | 0.521 |
| **Phe** | -1.0 | (1.0) | -0.8 | (1.0) | 0.710 | -0.9 | (0.9) | -0.2 | (1.2) | 0.122 |
| **Thr** | 0.8 | (1.0) | -1.0 | (1.8) | **0.002** | 0.9 | (1.4) | -0.2 | (1.2) | 0.037 |
| **Val** | -2.1 | (0.9) | -1.3 | (0.8) | 0.021 | -2.0 | (0.9) | -1.8 | (1.3) | 0.620 |

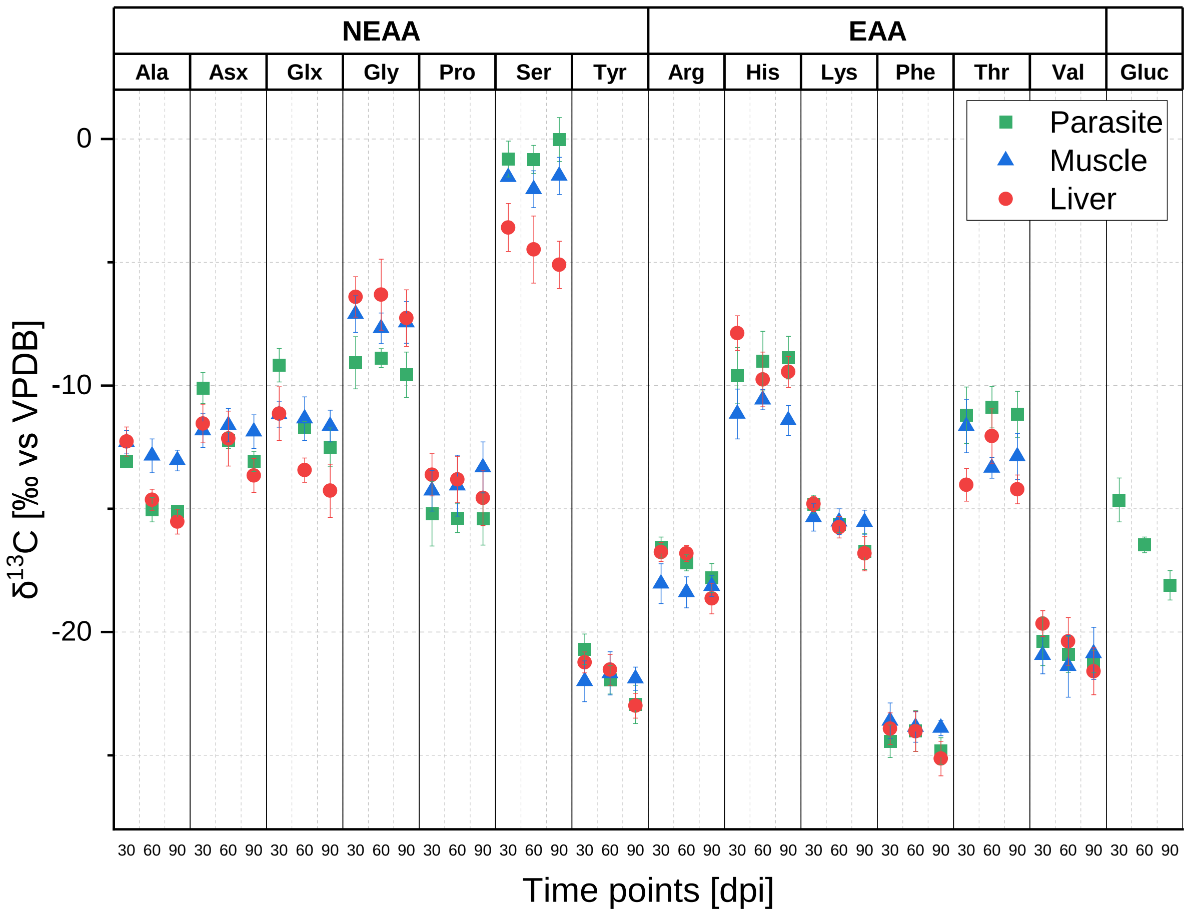


**Figure S1:** Average $\delta^{13}C$ values ± SD (n = 5) of individual AAs and glucose at 30, 60 and 90 dpi for host liver, muscle and parasite samples.

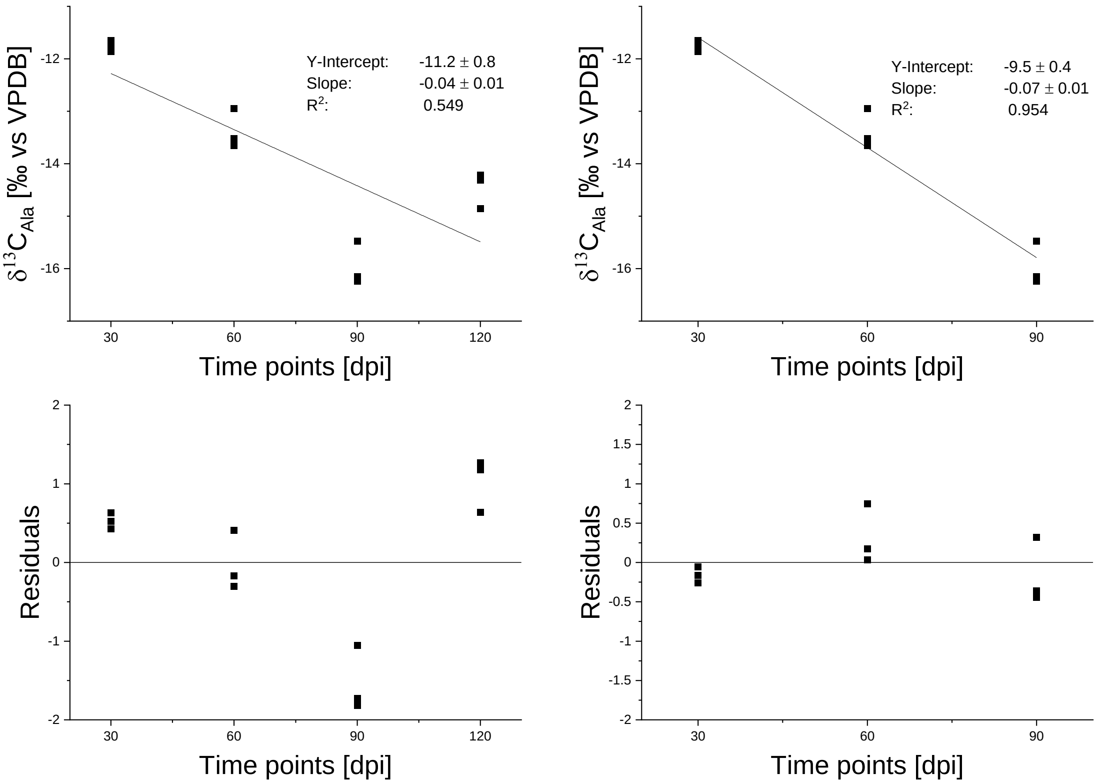


**Figure S2:** Changes of dietary $\delta^{13}C$ values over the first 90 days (right) of the experiment show a linear trend of decreasing $\delta^{13}C$ values, whereas changes of $\delta^{13}C$ over the whole 120 days of the experiment (left) results in a worse approximation by a linear fit due to the sudden increase in $\delta^{13}C$ values after 120 days. Top graphs show $\delta^{13}C$ values and linear fit with y-intercept, slope and correlation coefficient ($R^2$), while bottom graphs show the residuals. Data is only shown for Ala as an example and was taken from a previous study (Hesse et al. 2022) utilizing samples of dietary and uninfected control fish during the same infection experiment.

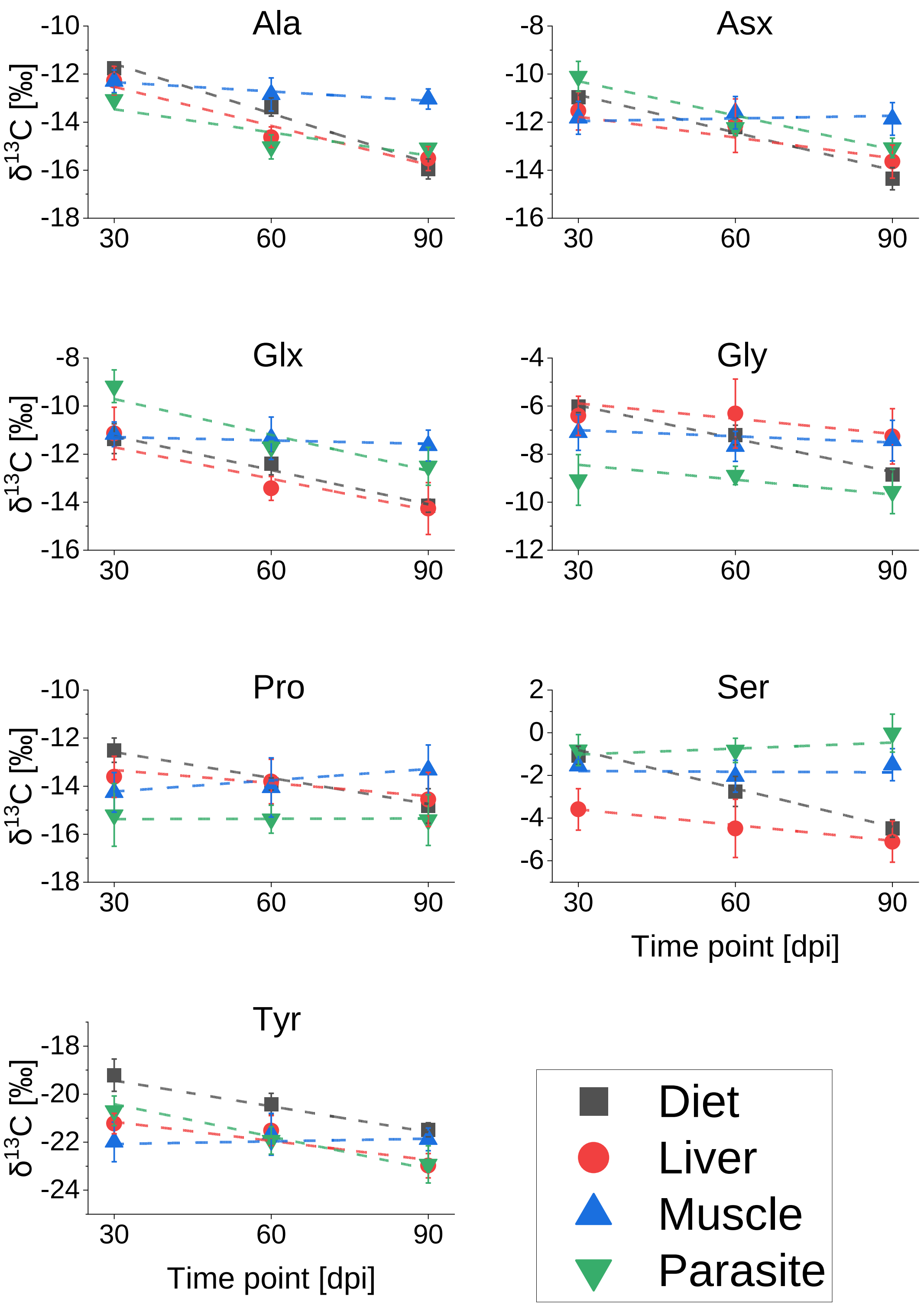


**Figure S3:** Linear regression of $\delta^{13}C$ values (‰ vs VPDB) ± standard deviations (whiskers) over 90 dpi from dietary, liver, muscle and parasite tissue of individual NEAAs. Data for dietary samples are taken from an earlier study (Hesse et al. 2022) and show a linear decrease of $\delta^{13}C$ values. Slopes of linear regression for liver and parasite tissue are negative for Ala, Asx, Glx and Tyr (one-way ANOVA).

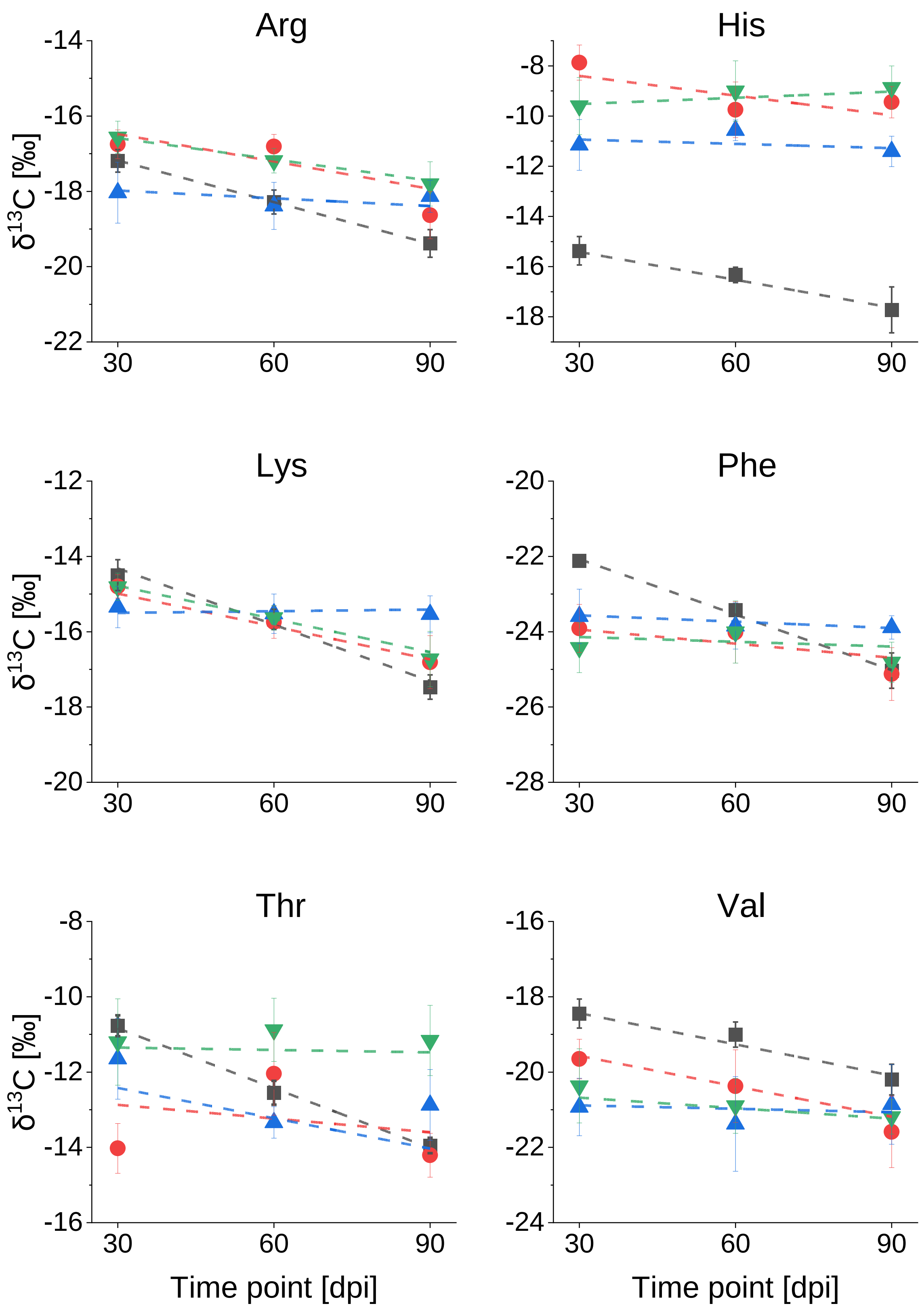


**Figure S4:** $\delta^{13}C$ values (‰ vs VPDB) ± standard deviations (whiskers) and linear regression curves over 90 dpi from dietary, liver, muscle and parasite tissue of individual EAAs. Symbols and colors are analogue to **Figure S3**.
